# Cytotoxic Lignan from the Non-Transformed Root Culture of *Phyllanthus amarus*

**DOI:** 10.3390/molecules20057915

**Published:** 2015-04-30

**Authors:** Barbara Sparzak, Mirosława Krauze-Baranowska, Anna Kawiak, Paweł Sowiński

**Affiliations:** 1 Department of Pharmacognosy with Medicinal Plants Garden, Medical University of Gdańsk, Gdańsk 80-210, Poland; E-Mail: bsparzak@gumed.edu.pl; 2 Department of Biotechnology, Intercollegiate Faculty of Biotechnology, University of Gdańsk and Medical University of Gdańsk, Gdańsk 80-822, Poland; E-Mail: anna.kawiak@biotech.ug.edu.pl; 3 NMR Laboratory, Faculty of Chemistry, Technical University of Gdańsk, Gdańsk 80-233, Poland; E-Mail: pawel.sowinski@pg.gda.pl

**Keywords:** *Phyllanthus amarus*, 7'-oxocubebin dimethylether, cytotoxic activity, *in vitro* culture

## Abstract

A new lignan from the non-transformed root *in vitro* cultures of *Phyllanthus amarus* was isolated. The structure of the compound was established on the basis of one- and two-dimensional NMR, as well as mass spectrometry data, as 7'-oxocubebin dimethylether (1,4-bis(benzo[*d*][1,3]dioxol-5-yl)-2,3-bis(methoxymethyl)butan-1-on). The non-transformed root cultures of *P. amarus* showed to be a selective source of this compound. The lignan revealed strong cytotoxic activity against HeLa cell line with an IC_50_ value of 3.8 µg/mL.

## 1. Introduction

*Phyllanthus amarus* Schum. and Thonn. (Euphorbiaceae) is widely distributed in tropical and subtropical regions of the world and has a long history in traditional therapeutic systems of Asia and South America as a medicinal plant [1]. The aerial parts of the plant are commonly used in the treatment of liver diseases, jaundice, intestinal infection and genitourinary disorders [1,2]. Due to its proven hepatoprotective and antiviral activity against the hepatitis B virus, *P. amarus* is one of the most intensively investigated species within the *Phyllanthus* genus [1,2]. The phytochemical study of *P. amarus* revealed the presence of lignans, flavonoids and hydrolysable tannins [1,3]. It has been reported that mainly lignans, next to ellagitannins, are responsible for the hepatoprotective and antihepatitis B activity of this plant [4,5,6]. The lignan complex of *P. amarus* consists of phyllanthin, hypophyllanthin, niranthin and nirtetralin, as the dominating constituents and heliobuphthalmin lactone, virgatusin and phyltetralin as the minor compounds [7,8,9,10]. The richest source of lignans is leaves, while stems, roots and fruits contain only small amounts of these compounds [11,12]. Because of a significantly low concentration of biologically active compounds, the roots of *P. amarus* are rarely investigated in terms of their chemical composition and pharmacological activity [13,14].

Several biotechnology studies concerning both hairy root and non-transformed root cultures of *P. amarus* have been reported [13,14,15]. The research revealed higher antihepatitis B activity of some *in vitro*-obtained biomasses in comparison to that of naturally occurring, leafy shoots [14]. Moreover, the results showed that the hairy root extract displayed linear concentration- and time-dependent cytotoxicity towards the MCF-7 cell line [15]. However, none of these studies provide detailed information regarding the chemical composition of *in vitro* cultivated roots of *P. amarus*.

The research presented herein describes the isolation, structure elucidation, and cytotoxic activity determination of an unknown lignan-type compound, which is selectively accumulated in non-transformed roots of *P. amarus* obtained *in vitro*.

## 2. Result and Discussion

Compound **1** was isolated as a white amorphous solid from the hexane fraction of ethanol extract from the non-transformed roots of *P. amarus* by preparative TLC. It gave a positive ESI pseudomolecular ion peak at *m/z* 401 [M+H]^+^ and adducts at 423 [M+Na]^+^ and 439 [M+K]^+^, suggesting the molecular weight to be 400. On the basis of MS data the molecular formula of **1** was assigned as C_22_H_24_O_7_.

**Figure 1:**
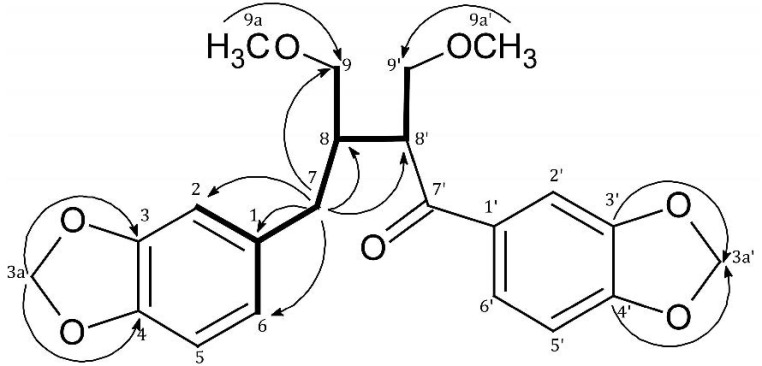
Selected COSY (Correlation Spectroscopy) and HMBC correlations of **1**.

The ^1^H-NMR data of **1** exhibited signals of two methylenedioxyl groups at δ 6.04 (2H, s, 3,4-OCH_2_O-) and δ 5.91 (2H, s, 3',4'-OCH_2_O-). In the ^13^C-NMR spectrum, characteristic signals for methylene carbon of the methylenedioxyl functions at δ 102.5 (C-3a) and δ 103.7 (C-3a') were observed. The position of methylenedioxyl groups was confirmed by the HMBC (Heteronuclear Multiple-Bond Correlation) correlation signals of C-3 and C-4 with protons at δ 6.04 as well as C-3' and C-4' with protons at δ 5.91 (Figure 1, Table 1).

Moreover, ^1^H-NMR spectrum of **1** showed signals of two methoxymethylene groups at δ 3.12, (1H, dd), 3.15 (1H, dd) and δ 3.61, (1H, dd), 3.72 (1H, t), one methylene group at δ 2.53 (2H, m) and two ABX systems for six aromatic protons in the range δ 6.61–7.42 (H-2, H-5, H-6 and H-2', H-5', H-6') (Table 1).

Two singlets at δ 3.20 (3H, s) and δ 3.17 (3H, s) in the ^1^H-NMR spectrum of **1** and chemical shifts of carbon atoms in the ^13^C- NMR spectrum at δ 59.2 and δ 59.5 displayed two aliphatic methoxy groups that were attached to C-9 and C-9' (Table 1). 

**Table 1:** 1D- and 2D-NMR experiments data of **1**.

Position	δ_H_ ( *J* in Hz)		δ_C_ (from HSQC)	HMBC	COSY
1	-		135.6	-	-
2	1H, 6.66 (d, 1.5)		110.8	C-7,C-6, C-3, C-4	H-6
3	-		149.0	-	-
4	-		147.3	-	-
5	1H, 6.74 d (7.8)		108.9	C-1, C-3, C-4	H-6
6	1H, 6.61 dd (7.8, 1.5)		123.6	C-2, C-4, C-7	H-2, H-5
7	2H, 2.53 m		36.4	C-1, C-2, C-6, C-8, C-8', C-9	H-2, H-6, H-8
8	1H, 2.15 m		43.1	C-1, C-7, C-9, C-7', C-8'	H-7, H-9, H-8'
9	2H,	3.12 dd (9.7; 4.9) 3.15 dd (9.7; 4.7)	72.5	C-7, C-8, C-8', C-9a	H-8
3a (3,4-OCH_2_O-)	6.04 s		102.5	C-3, C-4	-
9a (9-OCH_3_)	3.17 s		59.2	C-9	-
1'	-		134.1	-	-
2'	1H, 7.26 d (1.7)		109.2	C-3', C-4', C-6', C-7'	H-6’
3'	-		149.6	-	-
4'	-		153.1	-	-
5'	1H, 6.86 d (8.3)		108.8	C-1', C-3', C-4'	H-6'
6'	1H, 7.42 dd (8.3, 1.7)		125.9	C-2', C-4', C-7'	H-2', H-6'
7'	-		201.3	-	-
8'	1H, 3.78 ddd (8.8; 4.5; 5.7)		47.7	C-7, C-8, C-9, C-7', C-9'	H-8, H-9'
9'	2H,	3.61 dd (8.8; 4.4) 3.72 t (8.8)	73.1	C-8, C-8', C-7', C-9a'	H-8'
3a' (3',4'-OCH_2_O-)	5.91 s		103.7	C-3', C-4'	-
9a' (9'-OCH_3_)	3.20 s		59.5	C-9'	-

HSQC—Heteronuclear Single Quantum Correlation.

In the ^13^C-NMR spectrum, two methoxy-bearing methylene carbons appeared at δ 72.5 (C-9) and δ 73.1 (C-9'), that gave correlation signals with H-9a (δ_H_ 3.17/δ_C_ 72.5) and H-9a' (δ_H_ 3.20/δ_C_ 73.1), respectively, in the HMBC spectrum of **1** (Figure 1, Table 1).

The above-data, in comparison to literature data for lignans [16,17], suggests that compound **1** belongs to the group of diarylbutane derivatives with two methylenedioxyl rings.

The one methylene group carbon signal at δ 36.4 (C-7) was connected with the proton signal at δ 2.53 (H-7) in the HSQC spectrum of **1**, which led to conclusion that the carbon in C-7' position is substituted [16,17].

The signal at δ 201.3 was given for carbonyl group located at C-7' position. It was deduced from correlation signals present in the HMBC of **1** at δ_H_ 7.42/δ_C_ 201.3 (H-6'/C-7'), δ_H_ 7.26/δ_C_ 201.3 (H-2'/C-7'), δ_H_ 3.78/δ_C_ 201.3 (H-8'/C-7'), δ_H_ 3.61; 3.72/δ_C_ 201.3 (H-9'/C-7'), δ_H_ 2.15/δ_C_ 201.3, (H-8/C-7') (Table 1, Figure 1).

In the ROESY (Rotating-frame Overhause Effect Spectroscopy) spectrum of **1**, the following correlation signals for H-7 were observed—δ_H_ 6.6/δ_H_ 2.53 (H-2/H-7), δ_H_ 6.61/δ_H_ 2.53 (H-6/H-7), δ_H_ 2.53/δ_H_ 2.15) (H-7/H-8)—while there was a lack of correlations for H-8', H2' and H-6', which confirmed that there is no hydrogen atoms in C-7' position and support the conclusion that carbonyl group is placed in C-7'.

**Figure 2:**
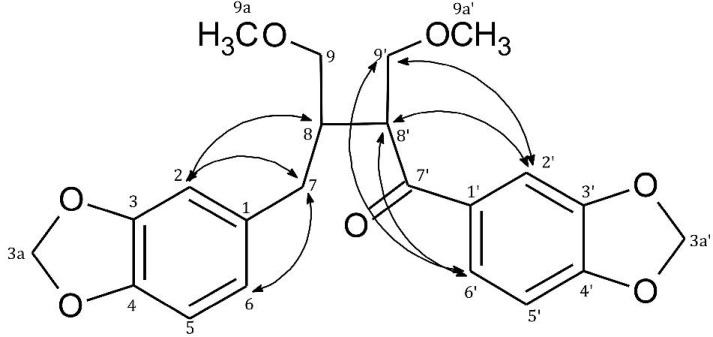
Selected ROESY correlations of **1**.

As a consequence of this fact, only the following correlation signals were observed in ROESY spectrum for H-2', H-6' and H-8'—δ_H_ 7.42/δ_H_ 3.78 (H-6'/H-8'), δ_H_ 7.42/δ_H_ 3.61; 3.72 (H-6'/H-9'), δ_H_ 7.26/δ_H_ 3.78 (H-2'/H-8') and δ_H_ 7.26/δ_H_ 3.61; 3.72 (H-2'/H-9') (Table 1, Figure 2). 

Two non-aromatic methine carbons appeared at δ 43.1 (C-8) and δ 47.7 (C-8'). The former signal at δ 43.1 showed long-range correlation with the proton signal at δ 2.53 (H-7).

The signals of two quaternary carbon atoms at δ 134.1 and 135.6 in the ^13^C-NMR spectrum were assigned to carbon atoms in C-1' and C-1 positions [10], due to the observed signals in the HMBC spectrum at δ_C_ 135.6/δ_H_ 2.53 (C-1/H-7), δ_C_ 135.6/δ_H_ 6.74 (C-1/H-5) and δ_C_ 134.1/δ_H_ 6.86 (C-1'/H-5') (Figure 1, Table 1).

Further correlation signals in the HMBC spectrum essential for elucidation of the structure of **1** were observed for H-7/C-8 (δ_H_ 2.53/δ_C_ 43.1), H-7/C-8' (δ_H_ 2.53/δ_C_ 47.7), H-7/C-9 (δ_H_ 2.53/δ_C_ 72.5), H-7/C-2 (δ_H_ 2.53/δ_C_ 110.8), H-7/C-6 (δ_H_ 2.53/δ_C_ 123.6), H-7/C-1 (δ_H_ 2.53/δ_C_ 135.6) (Table 1).

**Figure 3:**
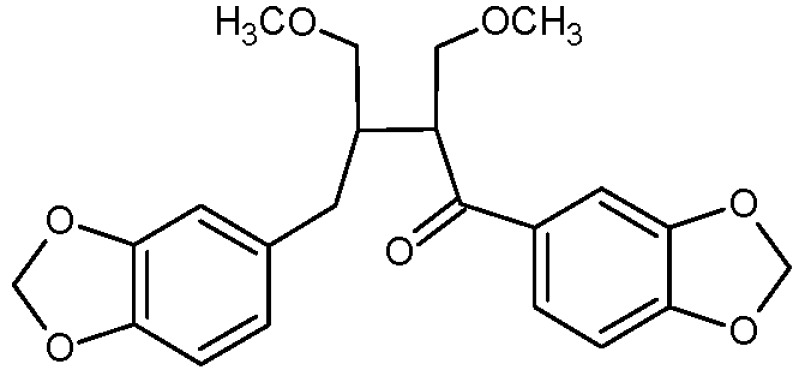
Chemical structure of **1**.

Regarding the results of the performed NMR and MS experiments, the structure of compound **1** was established as 1,4-bis(benzo[*d*][1,3]dioxol-5-yl)-2,3-bis(methoxymethyl)butan-1-on (7'-oxocubebin dimethyl ether) (Figure 3). 

Taking into consideration literature data it was determined that 7'-oxocubebin dimethyl ether is a new compound isolated from the plant kingdom. Until now, only one compound classified as a cubebin derivative, namely cubebin dimethyl ether, has been isolated from cell suspension culture of *P. niruri* [17].

The study of the cytotoxic activity towards three human tumor cell lines: HeLa, HTC116, MCF-7 included the methanol extract from non-transformed root culture of *P. amarus* and compound **1**. The human keratinocyte cell line (HaCaT) was used as a control cell line.

The extract from roots of *P. amarus* exerted cytotoxic activity only towards the HeLa cell line with an IC_50_ value of 85.0 ± 5.0 µg/mL. The study of 7'-oxocubebin dimethyl ether against the HeLa cell line showed significantly higher cytotoxic activity with an IC_50_ value of 3.8 ± 0.1 µg/mL (9.5 µM). The activity of the analyzed compound towards the HaCaT cell line was ten times lower (IC_50_ 38 ± 1.0 µg/mL). The results showed that 7'-oxocubebin dimethyl ether can be responsible for the cytotoxic activity of *P. amarus* root extract towards the HeLa cell line.

The study of Abhyankar *et al.* [15] showed that hairy roots of *P. amarus* exerted only marginal cytotoxic effects towards HeLa cells, contrary to their higher determined activity towards MCF-7 cells. However, the obtained IC_50_ value for the hairy root extract against MCF-7, after a 60 h treatment was low—200 µg/mL. According to the authors [15] the cytotoxic activity of the extract from hairy roots of *P. amarus* against MCF-7 cells results from the presence of amarone, which was detected only in root *in vitro* cultures while it was absent in the whole plant extract. However, the available literature data regarding this compound seems to be incomplete and unreliable [15,18].

Several reports on the cytotoxicity of *Phyllanthus* lignans concern the diarylbutane-type group of lignans—e.g., phyllanthin and niranthin. The cytotoxic potential of phyllanthin was determined towards different cancer cell lines, however the results showed that the obtained EC_50_ values exceeded the highest concentration of the tested compound (20 µg/mL) [19]. On the other hand, it was observed that phyllanthin enhances the cytotoxic response mediated by vinblastine in multidrug resistant (MDR) cells. Moreover, both lignans showed their potential as MDR reversing agents in myeloid leukemia, mainly due to their ability to synergize with conventional chemotherapeutics [20].

Another study showed that phyllanthin induced a dose- and time-dependent growth inhibition of HepG2 cells with the lowest EC_50_ value (10.16 ± 0.21 µg/mL/24.3 µM) obtained after 72 h of treatment [21].

Giridharan *et al.* [22] revealed that the 7'-hydroxy-3',4',5,9,9'-pentamethoxy-3,4-methylene dioxy lignan isolated from *P. urinaria* markedly inhibited the growth of HeLa, Hep2, MCF-7 and EL1 monocyte cell lines. However, there was no significant difference in the cytotoxic activity of the compound against the analyzed cell lines (IC_50_ values were not given).

Several papers report on the evaluation of the cytotoxic activity of butane-type lignans, both isolated from plants and synthesized [23,24,25,26]. A study concerning the cytotoxic effects of six dibenzylbutane-type lignans isolated from *Pycnathus angolensis* (*Myristicaceae*) or obtained by their derivatization showed that 4'-hydroxy-3,3',4-trimethoxylignan possesses the highest apoptosis-inducing activity towards human hepatoma HuH-7 cells. The compound displayed higher activity in comparison to the non-methoxylated derivatives. However, further methoxylation and acetylation resulted in decreased activity. The mechanism of action of 4'-hydroxy-3,3',4-trimethoxylignan was associated with the induction of caspase 3 activity [26]. The metoxylation-dependent cytotoxic activity of butane-type lignans was described by Lambert *et al.* [23]. The linear butane-type lignans were the most active against MCF-7 human breast cancer cells and the activity appeared to correlate positively with the number of *O*-methyl groups present on the molecule. This could be due to increased lipophilicity, which allows the compound to cross the plasma membrane of the cell [23]. On the other hand, the comparative study of the cytotoxic activity of synthesized meso-secoisolariciresinol and optically active secoisolariciresinols, revealed that only meso-secoisolariciresinol possessed cytotoxic activity towards mouse colon cancer, Colon-26 cells and MCF-7 cells, which indicates that the effect did not originate from the type of substituent but instead from its configuration [24].

Among the synthesized stereoisomers of the methoxybutane and fluorobutane type of 1,7-seco-2,7'-cyclolignans, the 9'-heptyl derivative showed the highest activity against HL-60 (IC_50_ = 3.7 µM) and HeLa cell lines (IC_50_ = 3.7 µM). The observed cytotoxic activity of the butane type 1,7-seco-2,7'-cyclolignans significantly reduced with the introduction of a hydroxyl group to the butane chain. The results of the research on the above mentioned lignan stereoisomers confirmed the positive correlation between the increase of hydrophobicity and cytotoxic effect of lignans [25].

Taking into consideration literature data, the presented results are the first showing high cytotoxic activity of a new biosynthesized diarylbutane-type lignan isolated from *P. amarus* roots obtained *in vitro*, towards the HeLa cell line.

## 3. Experimental Section

### 3.1. General

Preparative TLC was carried out on silica gel plates (TLC Si60 F_254_; Merck, Darmstadt, Germany) with hexane:ethyl acetate (3.5:6.5) (*v*/*v*) as mobile phase in ADC2 chamber (Camag, Numbrecht, Germany). Visualization of the TLC plates was performed under UV at λ = 254 and in daylight after spraying with 2% solution of H_2_SO_4_ in methanol followed by heating for 10 min in 110 °C.

NMR spectra were recorded on a Varian Unity Plus 500 MHz (Varian, Palo Alto, CA, USA) instrument at 500 MHz (for ^1^H) and 125 MHz (for ^13^C).

ESI mass spectra were recorded on TripleTOF^®^TM 5600+ mass spectrometer (AB SCIEX, Framingham, MA, USA) under the control of AB SCIEX Analyst TF 1.6 software.

### 3.2. Plant Material

The seeds used for the establishment of *in vitro* cultures of *Phyllanthus amarus Schum. & Thonn.* (*Euphorbiaceae*) were obtained from the Royal Botanical Garden in Brussels (Belgium) in 2009. Non-transformed root cultures of *P. amarus* were established by a classical method [18] from roots obtained from plants propagated *in vitro* on MS_0_ medium.

The root culture was propagated on ½SH medium supplemented with IBA 1.0 mg/L, with the concentration of macro and micro salts reduced by one-half [27].

The voucher specimen is kept in the herbarium of the Medicinal Plants Garden of the Medical University of Gdańsk (Poland).

### 3.3. Extraction and Isolation

Lyophilized and pulverized roots of *P. amarus* (50 g) obtained *in vitro* were exhaustively extracted with ethanol (5 × 500 mL) under a reflux condenser. The extract was filtered and evaporated under reduced pressure to give a dry residue, which was suspended in water (50 mL) and partitioned with hexane (9 × 50 mL). The hexane fractions were combined, concentrated and subjected to preparative TLC.

The hexane fraction was applied on the TLC Si60 F_254_ plates as 19 cm bands, 10 mm from the bottom edge of the plates. Plates were developed to a distance of 8.0 cm at room temperature. The silica gel containing compound **1** was scratched and extracted with methanol under a reflux condenser. The methanol extract was mixed with water and lyophilized.

### 3.4. Chemistry

*7'-Oxocubebin dimethyl ether* (**1**): white amorphous solid, (1,4-bis(benzo[*d*][1,3]dioxol-5-yl)-2,3-bis(methoxymethyl)butan-1-on, TLC Si60 F_254_, R_f_ = 0,76. ESI-MS (+): *m*/*z* = 401 [M+H]^+^, 423 [M+Na]^+^, 439 [M+K]^+^. M.W. 400. UV λ_max_ = 230, 279 nm, ${\left[\alpha \right]}_{D}^{25}$ = −40° (c = 0.2, MeOH), for ^1^H-NMR (CD_3_CN) and ^13^C-NMR (CD_3_CN) see Table 1). HRESI-qTOF-MS *m/z* [M+H]^+^ 401,1598 (calculated for C_22_H_25_O_7_^+^ 401,1595).

### 3.5. Cytotoxic Activity

All chemicals were purchased from Sigma-Aldrich (St. Louis, MO, USA).

The MCF-7 (human breast cancinoma) cell line was purchased from Cell Lines Services (Eppelheim, Germany), the HeLa (human cervical adenocarcinoma), HCT116 (human colorectal carcinoma) and HaCaT (human keratinocyte) cell lines were obtained from the Department of Microbiology, Tumor and Cell Biology, Karolinska Institute (Stockholm, Sweden). Cells were cultured in Dulbecco’s modified Eagle’s medium (DMEM) supplemented with 10% fetal bovine serum, 2 mM glutamine, 100 units/mL penicillin, and 100 μg/mL streptomycin. The medium for the HaCaT cell line was additionally supplemented with 4500 mg/L glucose. Cultures were maintained in a humidified atmosphere with 5% CO_2_ at 37 °C in an incubator (Heraceus, HeraCell).

The viability of the cell lines was determined using the MTT [(3-(4,5-dimethylthiazol-2-yl)-2,5-diphenyltetrazolium bromide) assay. The examined extract and compound were dissolved in DMSO, with 0.5% as the final concentration used in treatments. Cells were seeded in 96-well microtitre plates (5 × 10^3^ cells/well) and treated for 72 h with the test compounds. MTT (0.5 mg/mL) was added and the mixture incubated for 3 h at 37 °C following lysis of cells with dimethyl sulfoxide (DMSO). Optical density of the formed formazan solution was measured at 550 nm with a plate reader (Victor, 1420 multilabel counter) (Perkin Elmer, Turku, Finland). Experiments were carried out in triplicate (*n* = 3).

Dried and pulverized non-transformed root culture of *P. amarus* harvested on ½ SH medium supplemented with IBA 1.0 mg/L (2.0 g each) were extracted with methanol in boiling temperature (3 × 150 mL, 3 × 30 min). The obtained extracts were combined, filtrated and reduced under reflux condenser. The reduced methanol extract was mixed with water and lyophilized.

## 4. Conclusions

The non-transformed root *in vitro* cultures of *Phyllanthus amarus* is a selective source of new lignan compound—7'-oxocubebin dimethylether, which showed strong cytotoxic activity against HeLa cell line. It is noteworthy that our results are the first showing a tendency towards lignan production in root cultures of *P. amarus* [13,14,15].

## Supplementary Materials

ESI-MS and 1D- and 2D-NMR spectra of compound **1** are available as Supporting Information.

Supplementary materials can be accessed at: http://www.mdpi.com/1420-3049/20/05/7915/s1.
