# Supplementary material for: Cytotoxic Lignan from the Non-Transformed Root Culture of Phyllanthus amarus
Source: Molecules. 2015 Apr 30;20(5):7915–24. doi: 10.3390/molecules20057915 (PMC6272180; doi:10.3390/molecules20057915)
Supplement: Supplementary file 1 [file molecules-20-07915-s001.pdf]

Supplementary Materials

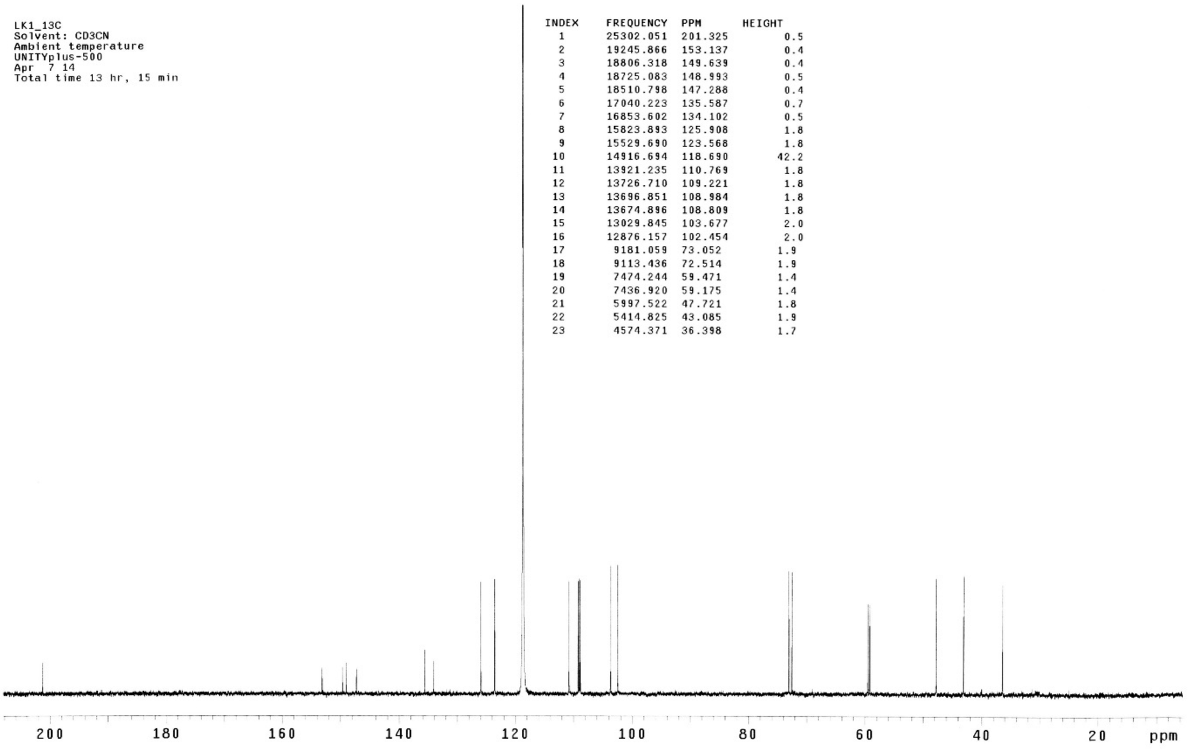

Figure S1. ^{13}C-NMR spectrum of compound **1**.

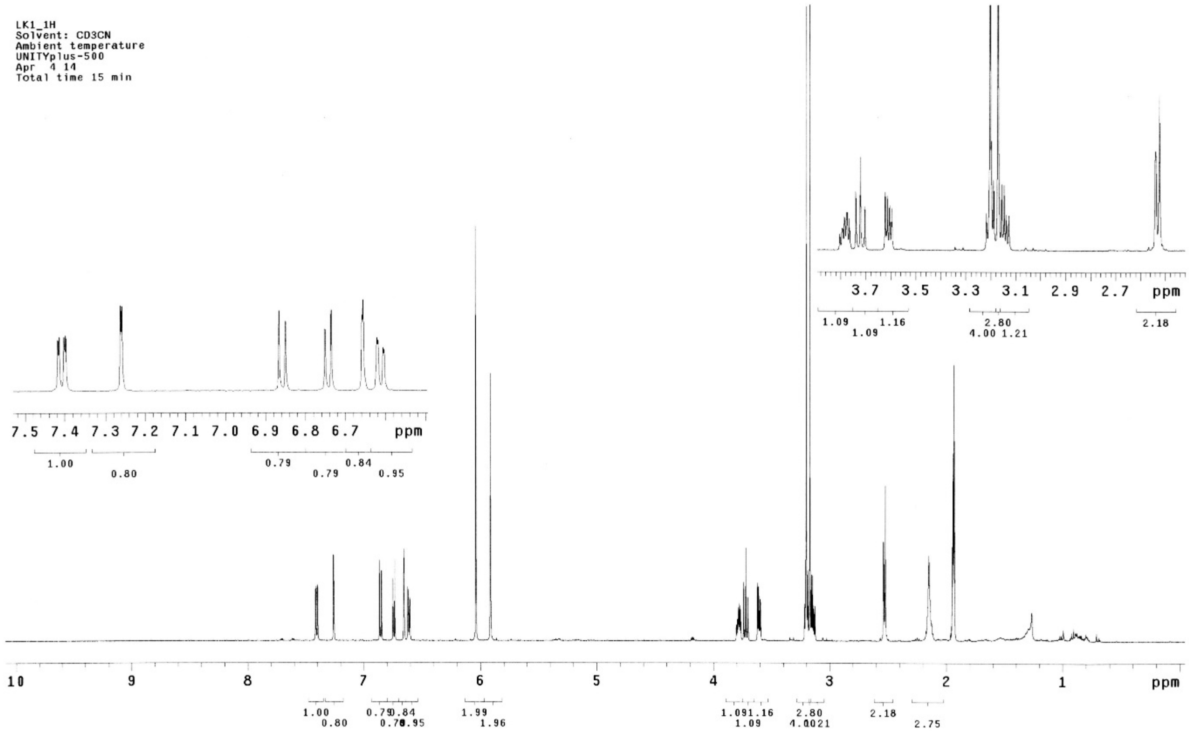

Figure S2. ^{1}H-NMR spectrum of compound **1**.

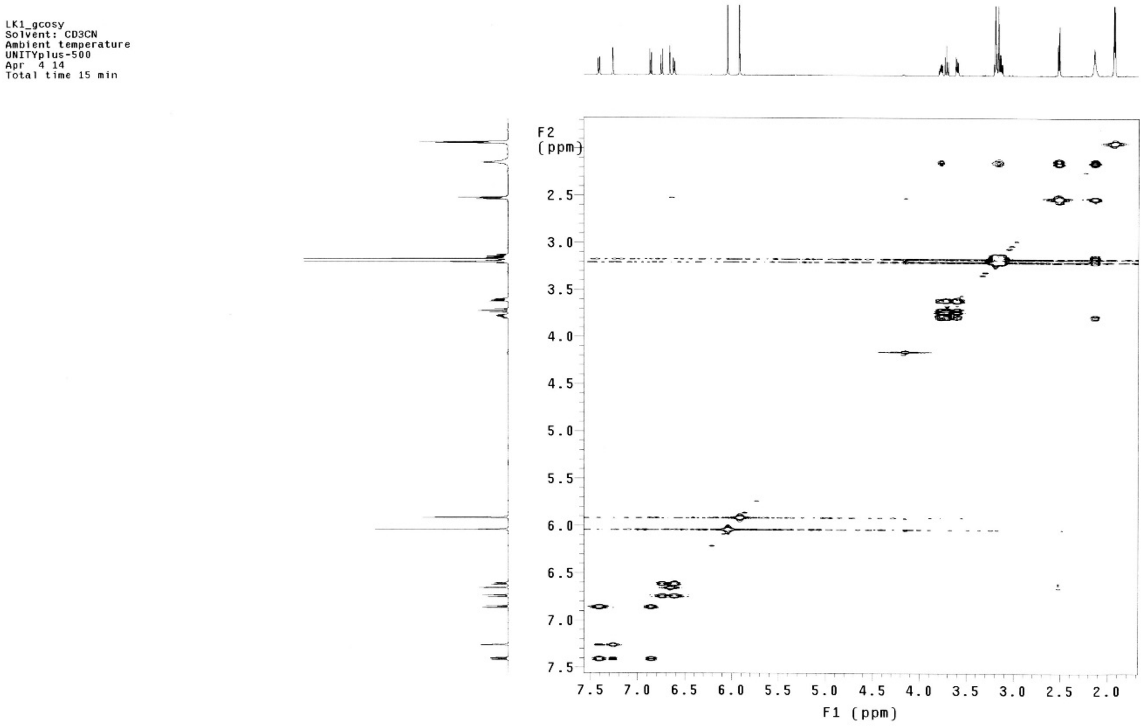

Figure S3. COSY spectrum of compound **1**.

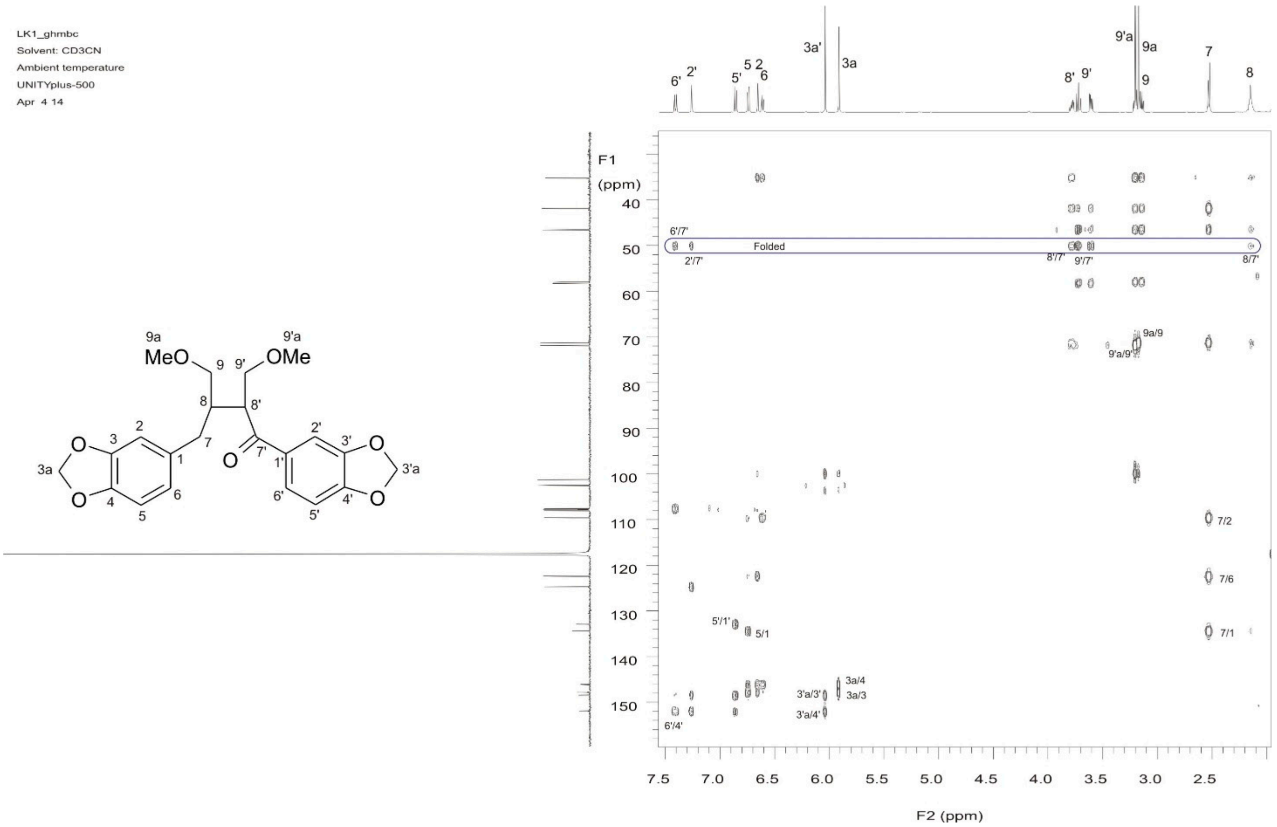

Figure S4. HMBC spectrum of compound **1**.

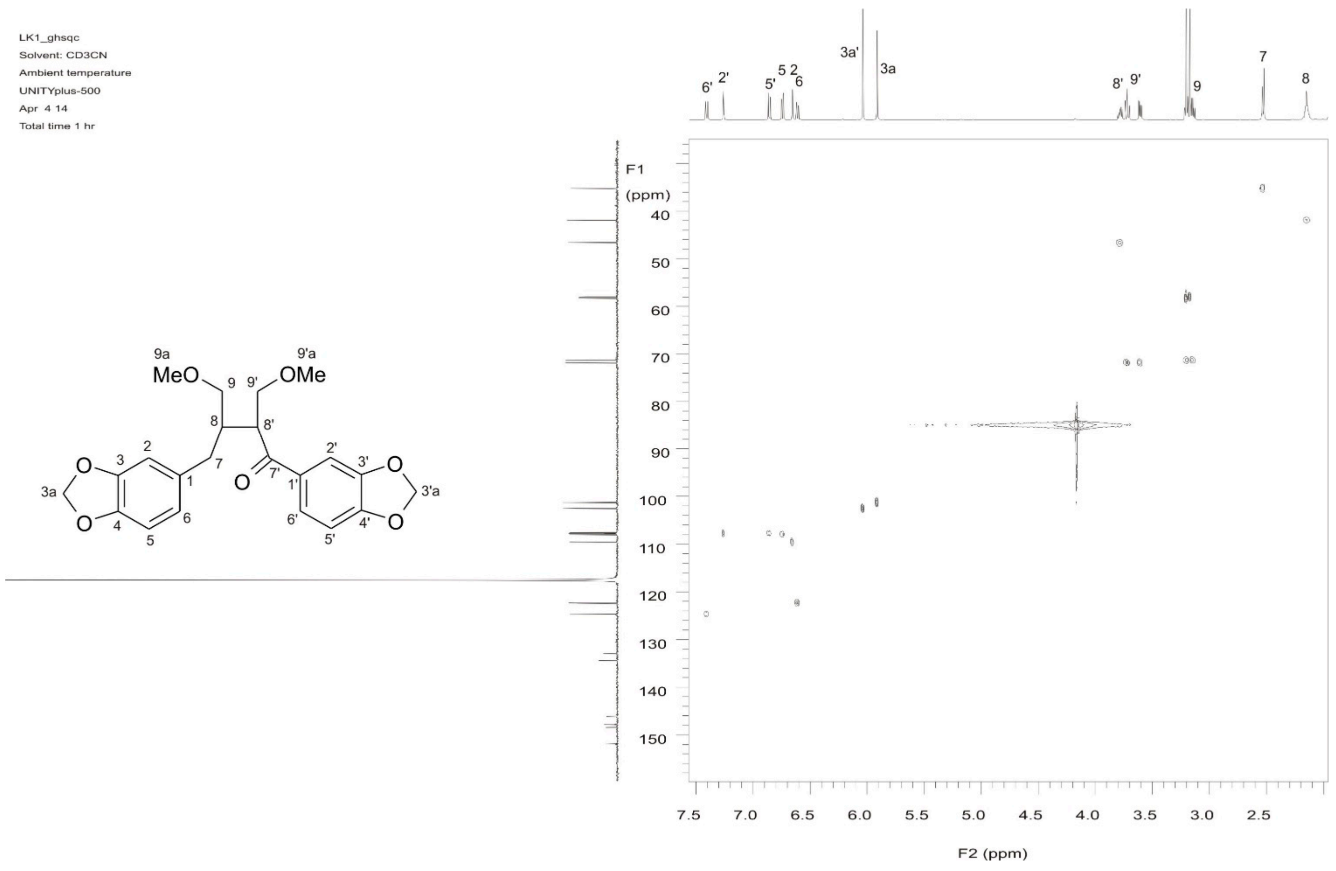

Figure S5. HSQC spectrum of compound **1**.

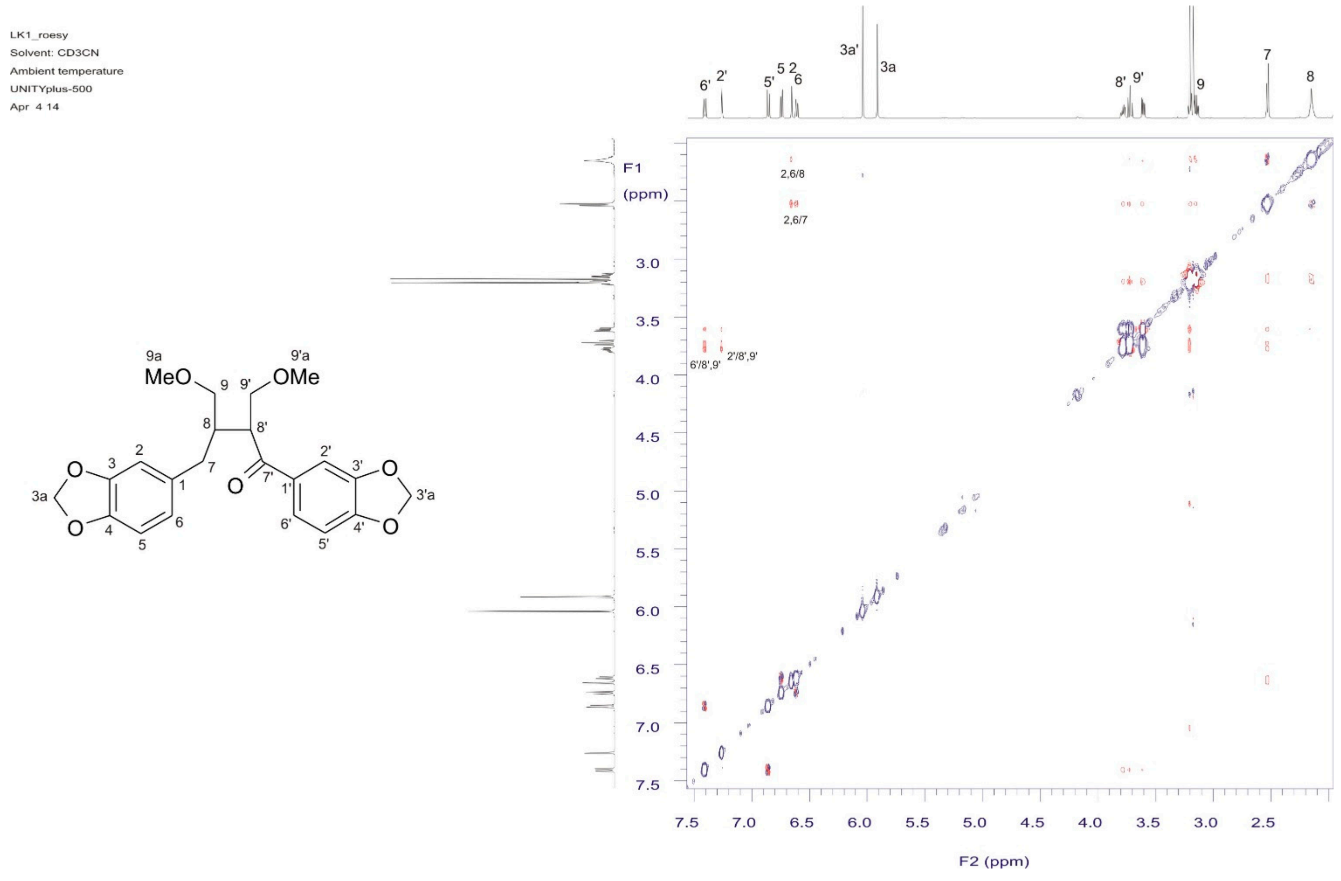

Figure S6. ROESY spectrum of compound **1**.

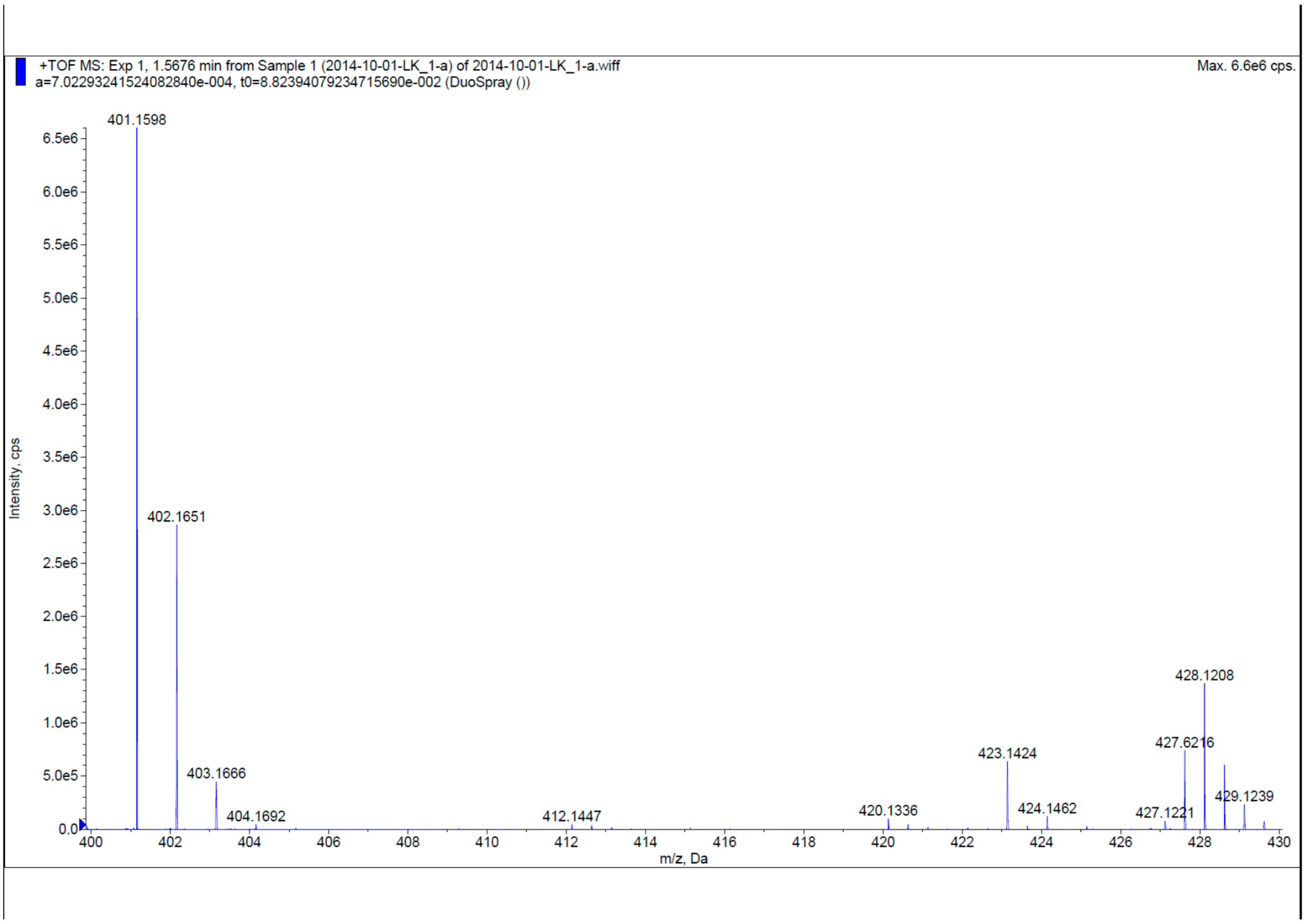

Figure S7. MS spectrum of compound **1**.

The dose-response curves for cytotoxicity data.

Cytotoxicity of methanol extract from non-transformed roots of *P. amarus* against HeLa cell line

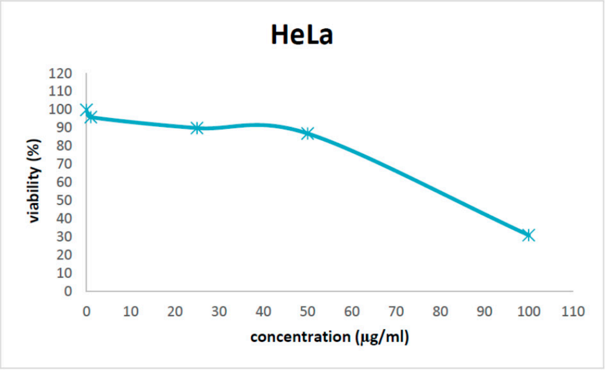

Cytotoxicity of compound **1** – 7'-oxocubebin dimethyl ether against HeLa and HaCaT cell lines.

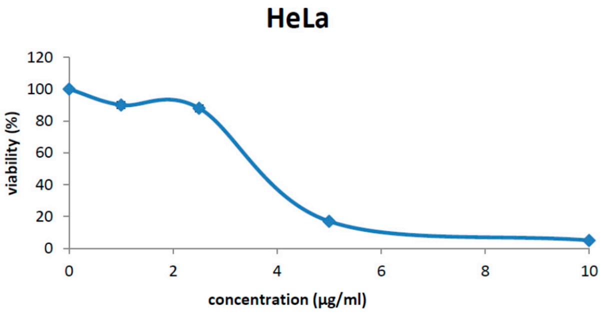

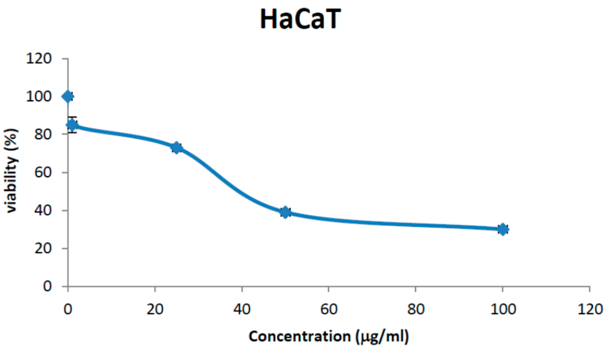

Figure S8. Dose-response curves for cytotoxicity of compound **1**.
